# Long-term representational costs of overloading working memory

**DOI:** 10.3758/s13423-025-02826-y

**Published:** 2026-02-19

**Authors:** Nathaniel R. Greene, Dominic Guitard, Alicia Forsberg, Nelson Cowan, Moshe Naveh-Benjamin

**Affiliations:** 1 Department of Psychological and Brain Sciences, Villanova University, 148 Tolentine Hall, 800 E. Lancaster Ave, Villanova, PA 19085 USA; 2 School of Psychology, Cardiff University, Cardiff, UK; 3 School of Psychology, The University of Sheffield, Sheffield, UK; 4 Department of Psychological Sciences, University of Missouri, Columbia, MO USA

**Keywords:** Working memory, Long-term memory, Gist, Aging

## Abstract

Can learning too much information at once impair long-term retention of its meaning? Emerging evidence suggests that encoding too many items into working memory (WM) limits subsequent long-term memory (LTM) retrieval of their details *and* gist. These findings highlight a boundary condition for theories positing relatively automatic gist encoding. But how expansive is this boundary? Experiment 1 shows that it extends to older adults, despite their generally enhanced reliance on gist memory. In two older adult samples (*n* = 40 each), LTM gist retrieval was reduced for objects encoded in sets exceeding WM capacity. Experiment 2 shows that this boundary holds even when retaining items in LTM is essential. Under intentional long-term learning, young (*n* = 81) and older (*n* = 40) adults’ LTM gist retrieval remained affected by overloading WM at encoding. Results invite leading memory theories to reconsider the universality of relatively automatic gist encoding.

**Supplementary Information:**

The online version contains supplementary material available at 10.3758/s13423-025-02826-y.

When attempting to retrieve a previous experience from long-term memory (LTM), success partly depends on how constrained working memory (WM) was at the time of its encoding. WM can maintain only a few items concurrently for ongoing cognitive operations (Cowan, 2001, 2017). Exceeding this capacity limit increases long-term forgetting. This WM-to-LTM bottleneck exhibits surprising consistency across the lifespan (Forsberg et al., 2021, Forsberg, Guitard, Adams et al., 2022a, Forsberg, Guitard, Greene et al., 2022b; Fukuda & Vogel, 2019) and is anticipated by dual-store (Atkinson & Shiffrin, 1968) and embedded processes (Cowan, 1988; Cowan et al., 2024) theories that view WM as the gateway through which new LTMs are formed.

But does overloading WM at encoding yield all-or-none or graded long-term forgetting? Can we retrieve from LTM the meaning (gist) of events encoded under supra-capacity conditions for which we cannot retrieve their specific details? These questions carry profound theoretical and practical implications for understanding attention’s role in encoding durable gist representations that persist over time (cf. Greene & Naveh-Benjamin, 2022b, 2023b). Fuzzy-trace theory (FTT; Brainerd & Reyna, 1990) assumes relatively automatic gist encoding in most situations,^1^ such that gist representations can be established under conditions of severely limited attention where verbatim (detailed) encoding is hampered. Supporting evidence shows that LTM gist retrieval is not as severely affected as LTM verbatim retrieval is by dividing attention with a concurrent task during encoding (Greene & Naveh-Benjamin, 2023b; Odegard & Lampinen, 2005; but see Greene & Naveh-Benjamin, 2022b, 2023b, for evidence that gist encoding still requires some, albeit reduced, commitment of attention). It is not strictly *necessary* that gist encoding be relatively automatic, and indeed it could be effortful in certain contexts involving the gradual accumulation of meaning over time (e.g., in extracting meaning from narratives when one’s attention is selectively oriented toward peripheral features of the text, like checking for spelling errors; Singer & Remillard, 2008). Yet effortful gist extraction could theoretically be limited to situations, like reading comprehension, where inferring the meaning of stimulus *i* is dependent on connecting it with stimulus *i* – 1. Under more static conditions where gist extraction for each stimulus is independent, the relatively automatic gist encoding view holds considerable sway. For example, in scene perception, we can more quickly build a conceptual representation of what the scene is about than a detailed, perceptual representation of specific scene elements (Oliva & Torralba, 2006; Potter, 1976; Tatler et al., 2003).

However, emerging evidence suggests that relatively automatic gist extraction breaks down when attention must be split among multiple to-be-encoded items. Borrowing from long-standing span manipulation procedures that have consistently demonstrated detrimental effects of supra-capacity encoding (e.g., on response latencies in judgment tasks; Baddeley & Hitch, 1974), Greene et al. (2024) tasked young adults with studying familiar objects (e.g., an apple) in sequences of six (supra-capacity) versus two (sub-capacity) items at a time. Under supra-capacity encoding, participants’ subsequent ability to retrieve from LTM not only the specifics (e.g., whether the apple was red or green) but also the gist (e.g., whether there was an apple or fruit at all) was impaired. Sometimes (e.g., when studying small-sized objects) overloading WM at encoding only limited LTM retrieval of gist but not specific representations, in complete opposition to FTT’s predictions. Do these findings reveal a boundary condition of FTT, one of the eminent theories of memory development? Here, we trace this boundary along two key lines to evaluate how expansive it is.

First, we ask whether this boundary extends to older adults, who preferentially rely on gist memory (Brainerd & Reyna, 2015; Greene & Naveh-Benjamin, 2020, 2023a; Koutstaal & Schacter, 1997; Tun et al., 1998). Despite age-related declines in WM (Light & Anderson, 1985; Wingfield et al., 1988) and episodic LTM (Zacks et al., 2000), recent evidence suggests that older adults’ LTMs are constrained by a WM bottleneck like that observed in younger adults (Forsberg, Guitard, Greene et al., 2022b; but see Bartsch et al., 2019). Yet the qualitative representational costs of this WM-to-LTM bottleneck might dissociate for young and older adults, even if one observed comparable LTM performance between the two groups. Older adults might rely on their expansive semantic memory to compensate for limitations in attention, enabling them to encode durable gist representations when WM is overloaded (cf. Greene & Naveh-Benjamin, 2024a). Then, overloading WM might only impede their long-term retention of specific, but not gist, representations, in line with FTT’s predictions. We test this possibility in Experiment 1.

Second, we ask whether this boundary recedes under conditions where participants deem it important to maintain information in LTM. Perhaps relatively automatic gist extraction broke down in Greene et al.’s (2024) study because LTM encoding was seemingly incidental (cf. McLaughlin, 1965): Participants had little reason to anticipate their LTM would be tested. Even if they automatically encoded each item’s gist when WM was overloaded, they may have dispensed with these representations when they were deemed no longer useful, following the immediate recognition tests at the end of each sequence. But if it was important to maintain these representations for a longer period of time, as is typical of students learning in a classroom, then perhaps overloading WM might not disrupt long-term gist retention. This would reinforce FTT’s relatively automatic gist extraction principle and could have applied implications in educational settings. We test this possibility in Experiment 2.

By mapping out the conditions under which learning too much information concurrently limits long-term gist retention, the present study places critical constraints on theories, like FTT, positing relatively automatic gist extraction. FTT has had an enormous influence on fields as diverse as decision making (Reyna, 2004), false memory (Brainerd & Reyna, 2005), and lifespan development (Brainerd & Reyna, 2015). Identifying when relatively automatic gist extraction breaks down, and when it holds, can reveal the boundaries where FTT’s reach ends.

## Experiment 1

Experiment 1 replicates and extends Greene et al.’s (2024) Experiment 1b, which yielded effects most opposed to FTT’s predictions: overloading WM at encoding impaired subsequent LTM retrieval of gist, but not specific, representations.^2^ We test whether these results extend to older adults, whose LTMs are generally more gist-based (Greene & Naveh-Benjamin, 2023a). If so, the boundary where FTT’s relatively automatic gist extraction principle (Brainerd & Reyna, 1990) breaks down extends across multiple age groups. If, however, overloading WM disproportionately limits older adults’ LTM retrieval of specific rather than gist representations, then FTT’s principle would still hold among individuals most prone to relying on gist memory.

### Method

#### Participants

We recruited 40 older adults (20 women, 20 men) ages 65 to 79 (*M* = 70.65, *SD* = 4.45) from Prolific, which has been shown to produce high-quality data (Peer et al., 2017), including with older adult samples (Greene & Naveh-Benjamin, 2022a). A plurality of participants had a bachelor’s degree (30%), followed by those with a GED (22.5%), a technical or associate’s degree (17.5%), and a high-school diploma, a master’s degree, or a doctorate degree (each comprising 10% of the sample). Participants were compensated at a rate of $15 (US) per hour of participation. We used the same eligibility criteria (with the exception of age) reported in Greene et al. (2024) to replicate the procedures as closely as possible. These were

1. Age between 65 and 85 years;
2. Native English speaker;
3. No self-reported history of language disorders, cognitive impairment, or dementia;
4. Fewer than nine units of alcohol (defined as 12 oz of beer, 5 oz of wine, or 1.5 oz of liquor) consumed per week;
5. Normal or corrected vision; and
6. A minimum approval rating of 90% based on prior submissions on Prolific.

We chose our sample size to match those reported in Greene et al. (2024). With *n* = 40, we achieved >80% power to detect effects of the same magnitude reported in Greene et al. (2024) of encoding set size on multinomial-processing-tree (MPT) model-based estimates of LTM gist retrieval (see online supplement). For comparative purposes, we re-plot the results of the 40 young adult participants (age: *M* = 26.18 years, *SD* = 2.79, range = 20–30) from Experiment 1b of Greene et al. (2024), as a baseline against which to compare the LTM representational effects of overloading WM in older adults. Because age group comparisons were not central to our hypotheses, we relegate those to the online supplement (see Table S1). Participants in all experiments provided their informed consent, and study procedures were approved by the Institutional Review Board at the University of Missouri and by the Ethics Committee at Cardiff University.

#### Materials and procedure

Stimuli were 377 objects, each sized to a resolution of 96 × 96 pixels, drawn from the stimulus sets of Brady et al. (2008). The 377 objects were randomly divided into 192 studied objects (96 per encoding set size condition), 176 unstudied objects used as either similar lures or new/different items, and 9 objects used in a practice phase. The experiment was programmed in PsyToolkit (Stoet, 2010, 2017) and administered online.

**Fig. 1:**
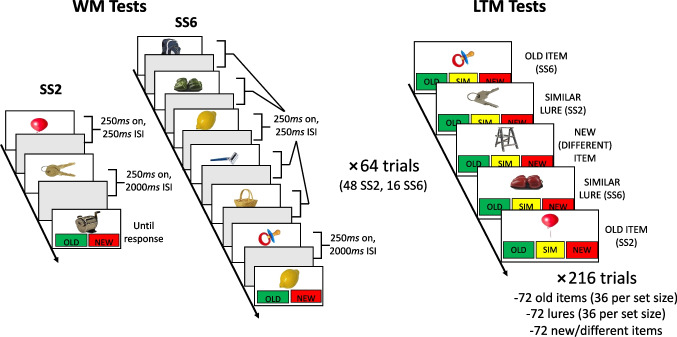
Experimental procedure. *Note.* During the first phase of the experiment, participants completed 64 working memory (WM) test trials. On each trial, either two (set size two, SS2) or six (set size six, SS6) objects appeared sequentially, for 250 ms per item with a 250-ms interstimulus interval (ISI) separating each item. Following the last item in the sequence, an additional 2,000 ms ISI occurred before the test probe. Half of the test probes were old items that had appeared in that sequence, and half were new items that had never appeared before in the experiment. After completing all WM trials, and following a 1-min interpolated activity task, participants completed 216 long-term memory (LTM) recognition tests evenly divided into 72 old items (36 from each set size), 72 similar lures to studied items (36 per set size), and 72 new/different items. No items were tested more than once in the experiment. (Color figure online)

Figure 1 summarizes the procedure. Participants studied objects in a random order in sequences of either two (SS2) or six (SS6) items. At the onset of the experiment, participants were informed that their memory would be tested following each study sequence and that they would later complete another memory task. However, they were not informed that this second memory task would be a delayed recognition test for items studied during the first part of the experiment. This information was not made available until the LTM test phase began.

During each WM trial, objects appeared sequentially for 250 ms each, with a 250 ms blank interstimulus interval (ISI) between objects, and an additional 2,000 ms blank ISI following the last item in the sequence. The stimulus onset asynchrony of 500 ms ensures sufficient time for consolidation of each item into WM up to a capacity limit (see Vogel et al., 2006), whereas longer encoding times might circumvent the capacity limit by allowing memorization or other strategies to play out. It also provides a sufficiently long time window for extracting the meaning of each visual item before the next item appears (e.g., Potter, 1976). In a follow-up experiment with an independent sample of 40 older adults, the objects on each trial appeared concurrently, with the display appearing for 500 ms in the SS2 condition and 1,500 ms in the SS6 condition (i.e., 250 ms per item). The procedures were otherwise identical and yielded comparable results (see online supplement, Fig. S1 and Table S2). At the end of each trial, a single probe appeared above two clickable response options labeled “old” or “new” in a green and red box, respectively. Participants had up to 60 s to make a response, and then pressed the space bar to initiate the next WM trial. Half of the trials featured an old item sampled randomly from the studied sequence, and half featured a new item completely unlike any object presented previously in the experiment. After completing 64 WM trials (48 at SS2 and 16 at SS6), participants completed an arithmetic verification task for 60*s* as an interpolated activity between the WM and LTM testing phases. Participants indicated whether each presented arithmetic problem was solved correctly (e.g., 2 × 4 + 3 = 11?) or incorrectly (e.g., 3 × 2 + 6 = 11?) by clicking one of two response options appearing below each problem.

Finally, participants completed delayed LTM recognition tests for the sequences studied during the first part of the experiment. The format of these LTM tests followed the simplified conjoint recognition procedure (Stahl & Klauer, 2008): three types of memory probes (old items, similar lures, and novel foils) were factorially crossed with three response options (“old,” “similar,” and “different”). Old items (targets) were drawn from both SS2 and SS6 sequences with the only constraint being that they were not previously used as test probes in the WM tests (i.e., LTM targets were *untested* studied items from the WM trials). Similar lures and novel foils never appeared before in the experiment. However, similar lures belonged to the same conceptual category as untested items from SS2 and SS6 sequences (see Fig. 1 for examples) and thus shared the same gist but not specific representations as these studied items. Novel foils were drawn from categories not previously appearing in the experiment and thus shared neither a common specific nor gist representation with any studied item. Participants clicked one of three responses appearing below each probe—“old,” “similar,” or “new/different” in a green, yellow, and red box, respectively—within 60 s and then pressed the space bar to initiate the next LTM trial. There were 216 LTM test trials, with 72 targets (36 per set size condition), 72 similar lures (36 lures per set size condition), and 72 new foils.

#### Analyses

By including a WM recognition test following each studied sequence, we were able to estimate, based on participants’ accuracy on these tests, WM capacity (*k*) using a hierarchical Bayesian WM capacity model to derive group- and individual-level estimates of *k* (see online supplement for details). This gave us an indication of approximately how many items in the SS2 and SS6 conditions participants could reasonably maintain in WM on each trial. These insights were critical for assessing whether we satisfactorily created a condition (SS6) where WM was overloaded at the time of encoding.

**Fig. 2:**
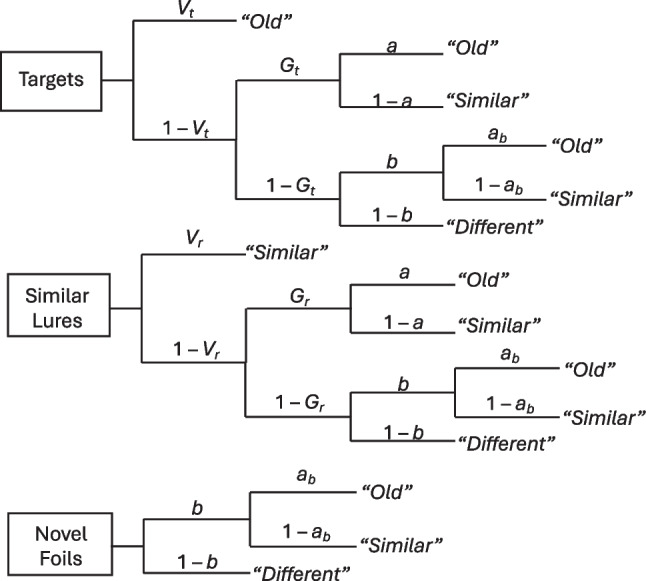
Simplified conjoint recognition multinomial-processing-tree (MPT) model. *Note.* Given a memory probe (boxes on the left), participants’ responses are modeled to arise from the contributions of verbatim/specific and gist memory retrieval and guessing. *V*_*t*_ and *V*_*r*_ denote the probabilities of retrieving a studied item’s verbatim (specific) representation when shown that item (a target) or a related lure, respectively. Verbatim retrieval leads to correct identification of targets as “old” and lures as “similar.” When verbatim retrieval fails, the participant may still be able to retrieve the studied item’s gist with probabilities *G*_*t*_ for targets and *G*_*r*_ for lures. Gist retrieval leads to an uncertainty state of whether the probe is a target or lure, so the participant must guess whether the probe is “old” (with probability *a*) or “similar” (with probability 1 – *a*). When both verbatim and gist retrieval fail, or when the probe does not share a similar gist with studied items (as in the case of novel foils), the participant might still decide that the probe *could be* a target or lure with probability *b*; otherwise, they respond “new/different.” Parameter *a*_*b*_ measures the bias to guess “old” in cognitive state *b*. Constraining *a*_*b*_ = *a* assumes the same bias to guess “old” in gist and non-gist retrieval states; otherwise, this bias is free to vary across these states

To address whether overloading WM at encoding impaired subsequent LTM gist retrieval, we used the MPT model from the simplified conjoint recognition paradigm (Stahl & Klauer, 2008; see Fig. 2) to estimate the probabilities of LTM specific and gist retrieval for items encoded under SS2 versus SS6 conditions. The model aims to explain how participants’ observed recognition responses to targets, similar lures, and novel foils arise from unobservable cognitive processes (verbatim and gist memory retrieval) and guessing. This is reflected in the branching structure in Fig. 2, where, at each node, a given process succeeds with probability *s* and fails with probability 1 – *s*. Summing the pathways leading to observed response *j* (e.g., “old”) to memory probe *i* (e.g., targets) maps the joint contributions of each latent process to *j*. Figure 2 caption provides a walk-through of the model.

We fit the model to the LTM recognition data using a hierarchical Bayesian latent-trait specification in the *TreeBUGS* package for R (Heck et al., 2018; see online supplement for technical details). We allowed parameters *V*_*t*_, *G*_*t*_, and *G*_*r*_ to vary by set size, but due to technical constraints, *V*_*r*_ was set equal across set size conditions. Despite this constraint, we were able to test our core hypothesis about whether WM set size affected LTM gist retrieval by asking whether estimates of the gist memory parameters (*G*_*t*_ and *G*_*r*_) were lower in the SS6 than SS2 conditions. To test for these effects, we subtracted the group-level posterior samples of each gist memory parameter (*G*_*t*_ and *G*_*r*_), along with those of *V*_*t*_, in the SS6 condition from the posterior samples of the respective parameter in the SS2 condition (e.g., Δ*G*_*t*_ = ${G}_{t}^{SS2}$ – ${G}_{t}^{SS6}$). We concluded that there was credible evidence for a WM set size effect if the 95% Bayesian credible interval (CI) of the resulting difference score Δ excluded 0 (cf. Smith & Batchelder, 2010). If the 95% CI of Δ encompassed 0, we remained agnostic as to whether a difference existed, akin to obtaining *p* > .05 in null hypothesis significance testing.

### Results

**Fig. 3:**
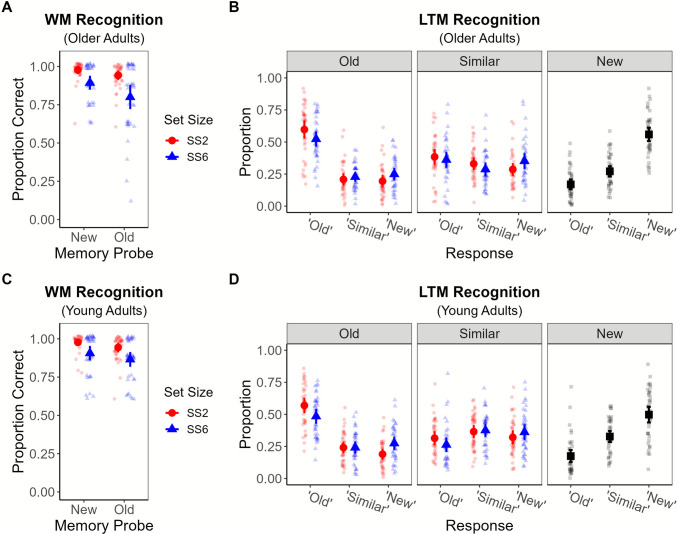
WM and LTM recognition results in Experiment 1. *Note.* Top row depicts the working memory (WM; **A**) and long-term memory (LTM; **B**) results for the older adults in Experiment 1, while the bottom row replots the WM (**C**) and LTM (**D**) data of young adults from Experiment 1b of Greene et al. (2024). WM results (**A** and **C**) depict the proportion of correct responses to old and new probes at each set size (SS2 and SS6). LTM results (**B** and **D**) depict the proportions of each response (“old,” “similar,” and “new”) to each probe (old, similar, and new items), with responses to old items and similar lures separated by the set size condition. Group means of each condition appear as large, bold shapes, with individual participants’ means underlaid as faded shapes. Error bars represent 95% confidence intervals of the means. (Color figure online)

Figure 3A and B depict older adults’ WM and LTM recognition performance, respectively. For comparative purposes, Fig. 3C and D show the recognition performance of young adults in the matched experiment by Greene et al. (2024). Older adults exhibited high WM recognition accuracy, but their memory discrimination, assessed via the bias-corrected nonparametric *A′* statistic (see Stanislaw & Todorov, 1999), was poorer in the SS6 (*M* = 0.91, *SD* = 0.08) than SS2 (*M* = 0.98, *SD* = 0.03) condition, *t*(39) = 5.48, *p* < .001. Furthermore, whereas they could reasonably maintain both items in WM for SS2 sequences, with a group-level posterior mean of *k* = 1.89, 95% CI [1.83, 1.95],^3^ their WM was overloaded by at least one item in the SS6 condition, *k* = 4.65, 95% CI [4.33, 4.94]. Similar results were reported previously with the young adult sample: SS2 *k* = 1.91, 95% CI [1.84, 1.98]; SS6 *k* = 5.10, 95% CI [4.83, 5.34] (see Greene et al., 2024, Experiment 1b).

In the LTM tests, older adults were more accurate at identifying targets as “old” if they were studied in SS2 sequences (*M* = 0.60, *SD* = 0.03) than SS6 sequences (*M* = 0.52, *SD* = 0.03), *t*(39) = 3.75, *p* < .001. However, their accuracy in identifying lures as “similar” did not significantly differ for lures to SS2 items (*M* = 0.33, *SD* = 0.02) and lures to SS6 items (*M* = 0.29, *SD* = 0.02), *t*(39) = 1.87, *p* = .069. Nonetheless, as listed in Table 1, older adults were less likely to retrieve the gist of a studied item in response to a lure (parameter *G*_*r*_) when WM was overloaded at the time the item was studied (i.e., ${G}_{r}^{SS6}<{G}_{r}^{SS2}$). That is, the 95% CI of the WM set size effect Δ*G*_*r*_ excluded 0. The 95% CI for Δ*G*_*t*_ encompassed 0 (see Table 1), so we remained agnostic about the presence of a WM set size effect on LTM gist retrieval for targets in this sample of older adults. Directionally, the effect hypothesis (${G}_{t}^{SS2}>{G}_{t}^{SS6}$) was favored in 94.8% of posterior samples, just shy of a one-tailed effect criterion of 95%. In the follow-up experiment using simultaneous presentation at encoding, we detected credible evidence for a WM set size effect on older adults’ LTM estimates of *G*_*t*_ and *G*_*r*_; both gist parameters were lower in the SS6 than SS2 condition (see Table S2 in the online supplement).

**Table 1:** WM set size effects on LTM retrieval of specific and gist representations in Experiment 1

Parameter	Age	SS2	SS6	Δ(SS2 – SS6)	*p*(SS2 > SS6)
*V*_*t*_	Old	0.26 [0.12, 0.39]	0.20 [0.08, 0.30]	0.07 [−0.06, 0.19]	0.853
*G*_*t*_	Old	0.54 [0.42, 0.65]	0.43 [0.33, 0.53]	0.10 [−0.02, 0.23]	0.948
*V*_*r*_	Old	0.03 [0.00, 0.07]	= SS2	—	—
*G*_*r*_	Old	0.46 [0.39, 0.52]	0.36 [0.29, 0.44]	0.09 [0.02, 0.17]*	0.991
*V*_*t*_	Young	0.25 [0.03, 0.40]	0.26 [0.09, 0.36]	−0.01 [−0.17, 0.12]	0.462
*G*_*t*_	Young	0.44 [0.25, 0.61]	0.18 [0.03, 0.38]	0.26 [0.06, 0.44]*	0.994
*V*_*r*_	Young	0.04 [0.00, 0.10]	= SS2	—	—
*G*_*r*_	Young	0.29 [0.20, 0.38]	0.18 [0.10, 0.27]	0.11 [0.03, 0.19]*	0.997

Multinomial-processing-tree (MPT) model-based estimates of long-term memory (LTM) specific/verbatim (*V*_*t*_ and *V*_*r*_) and gist (*G*_*t*_ and *G*_*r*_) retrieval in response to targets (*V*_*t*_ and *G*_*t*_) or related lures (*V*_*r*_ and *G*_*r*_) for items studied in working memory (WM) sequences of two (SS2) or six (SS6) items. Estimates are given as the group-level posterior means [95% Bayesian credible interval (CI)]. Older adults’ estimates of the response bias parameters (see Fig. 2) were: *a* = 0.69 [0.58, 0.80], *b* = 0.43 [0.37, 0.49], *a*_*b*_ = 0.35 [0.28, 0.42]. Young adults’ estimates of verbatim and gist retrieval are listed for comparative purposes; see Table 6 in Greene et al. (2024) for their estimates of response bias. * denotes that the 95% CI of the posterior difference score Δ excluded 0. *p*(SS2 > SS6) gives the proportion of posterior samples for which the specified parameter was estimated to be higher in the SS2 than the SS6 condition

We failed to detect a credible WM set size effect on older adults’ LTM verbatim retrieval for targets (i.e., the 95% CI of Δ*V*_*t*_ encompassed 0); this result replicated in our follow-up experiment (see Table S2 in the online supplement). Overall, results with older adults mostly replicated those obtained previously with younger adults (Greene et al., 2024, Experiment 1b), listed in the bottom half of Table 1. Given that the MPT model assumes that gist retrieval occurs *conditionally* on verbatim retrieval failing (see Fig. 2), the set size effects on the gist parameters in Table 1 and Table S2 could be viewed as evidence for a somewhat more all-or-none type of remembering of information if it was encoded while WM was overloaded. That is, there is apparently little, if any, difference in the probability of retrieving from LTM the specific details of an item encoded under within-capacity (SS2) versus supra-capacity (SS6) conditions. Yet if an individual, whether a young adult or an older adult, fails to retrieve these specifics, they are less likely to also retrieve the item’s gist if it was encoded in supra-capacity conditions. In addition, as reported in Table S1 in the online supplement, older adults were *more* likely than younger adults to retrieve a studied item’s gist in response to a lure (i.e., ${G}_{r}^{Young}<{G}_{r}^{Old}$) at both set sizes. Yet the same WM set size effect on *G*_*r*_ was obtained in both age groups (Table 1), suggesting that the effect of overloading WM at encoding on LTM gist retrieval operates similarly in groups that differ in their overall reliance on gist memory.

## Experiment 2

Results of Experiment 1 add to a growing deck of cards stacked against FTT’s relatively automatic gist extraction principle (cf. Greene et al., 2024; Ricker & Hardman, 2017). Yet this deck is almost exclusively composed of studies in which LTM encoding was incidental. Perhaps individuals do encode each item’s gist when WM is overloaded, but they discard these representations when it is deemed no longer useful to maintain them (e.g., after the immediate recognition test following each study sequence in the previous experiments). We tested this possibility by informing participants that their memory for items from each sequence would also be tested at the end of the experiment. If intentional learning for long-term retention eliminates the encoding set size effect on LTM gist retrieval, then FTT’s principle would still hold.

### Method

#### Participants

We recruited an additional 40 older adults (18 women, 22 men) ages 65 to 83 (*M* = 69.93, *SD* = 4.45) and 81 younger adults (33 women, 45 men, three gender nonbinary or nondisclosed) ages 18 to 30 (*M* = 24.17, *SD* = 3.12) from Prolific. Initially, we recruited 40 young adults, but we doubled the sample size because the evidence for an effect of WM set size on LTM gist retrieval was inconclusive at an *n* of 40 (see Table S3 in the online supplement).^4^ Because our MPT model hypothesis tests cannot test for a *null effect*, we deemed it important to test whether evidence for an effect would emerge under a larger sample size. Bayesian statistical inferences are robust to optional stopping (Rouder, 2014). Eligibility criteria were as specified in Experiment 1, except we restricted the age range for young adults to 18 to 30 years, as in Greene et al. (2024). In addition, participants were ineligible to participate if they previously participated in another of the experiments of the present study or those in Greene et al. (2024). The composition of educational attainment across the two age groups was mostly comparable: high-school diploma or GED (older adults: 30%; young adults: 24.7%); Associate’s or technical degree (older adults: 12.5%; young adults: 11.1%); bachelor’s degree (older adults: 50%; young adults: 40.7%); and a graduate-level degree (older adults: 7.5%; young adults: 22.2%).

#### Materials, procedure, and analyses

There was one key procedural difference from Experiment 1. At the start of the experiment, participants were informed that the second memory test, occurring at the end of the experiment, would be a delayed recognition test for items studied during the first part of the experiment. Thus, they were instructed to prioritize studying the items both for immediate testing at the end of each sequence *and* for delayed (LTM) testing to occur after all the sequences were studied. All other procedures were identical to Experiment 1.

### Results

**Fig. 4:**
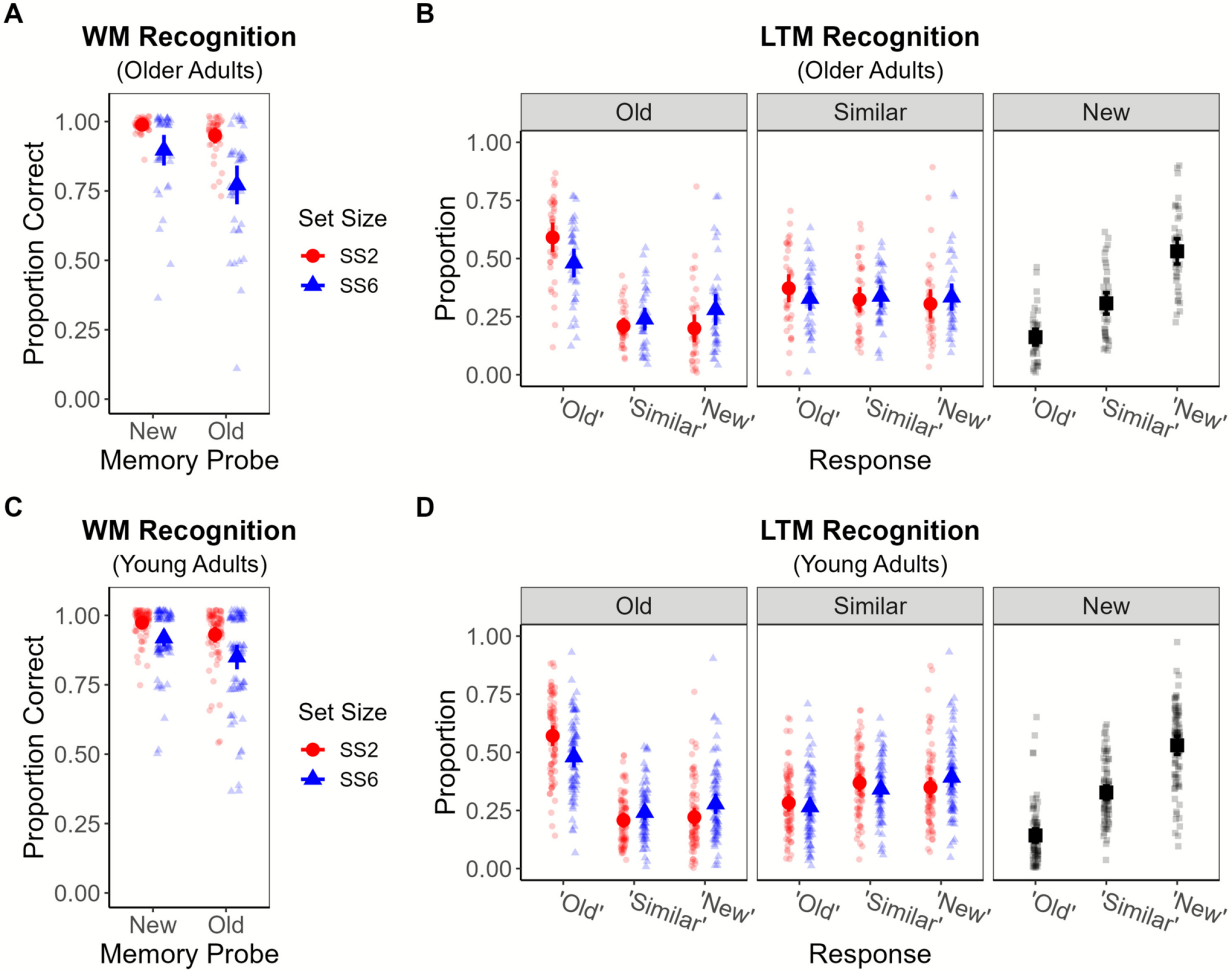
WM and LTM recognition results in Experiment 2. *Note.* Working memory (WM; **A**, **C**) and long-term memory (LTM; **B**, **D**) recognition accuracy plotted for older adults (top row) and young adults (bottom row) in Experiment 2. See Fig. 3 caption for further details. (Color figure online)

WM and LTM recognition accuracy are summarized in Fig. 4, with strikingly similar results across groups. Indeed, in the WM tests, memory discrimination (*A′*) was poorer in the SS6 than SS2 condition (see Table 2) among older adults, *t*(39) = 5.16, *p* < .001 and young adults, *t*(80) = 6.73, *p* < .001, alike. The results of a 2 (age) × 2 (set size) mixed analysis of variance (ANOVA) on WM *A′* also revealed a significant effect of set size, *F*(1, 119) = 76.53, *p* < .001, and a significant interaction, *F*(1, 119) = 12.05, *p* < .001, which was driven by a significant Bonferroni-adjusted age difference (young > old) in SS6 *A′*, *t*(119) = 2.65, *p*_adjusted_ = .009. In both groups, WM was overloaded by at least one item in the SS6 condition (see Table 2).

**Table 2:** WM results for Experiment 2

	Memory discrimination (*A′*)		WM Capacity (*k*)	
Age Group	SS2	SS6	SS2	SS6
Old	0.98 (0.02)	0.89 (0.11)	1.92 [1.86, 1.99]	4.48 [4.16, 4.78]
Young	0.97 (0.04)	0.93 (0.06)	1.88 [1.83, 1.93]	5.02 [4.82, 5.20]

*A′* is a nonparametric signal detection metric of memory discrimination, with a typical range from 0.5 (chance) to 1.0 (perfect discrimination). Values of *A′* are mean (*SD*). *k* represents the number of items out of *N* that can be held in working memory (WM). Values of *k* correspond to the group-level posterior mean [lower 2.5^th^ percentile, upper 97.5^th^ percentile] from a hierarchical Bayesian model. SS2 = set size two; SS6 = set size six

For the LTM tests, we conducted separate 2 (age) × 2 (set size) mixed ANOVAs on the proportion of correct responses to old items (*p*(“old”)) and similar lures (*p*(“similar”)). For the former, accuracy was lower for SS6 items, *F*(1, 119) = 67.88, *p* < .001, but we did not detect significant age or interaction effects, both *F*(1, 119) < 0.67, *p* > .416. For similar lures, neither main effect was significant, both *F*(1, 119) < 1.14, *p* > .289, but there was a marginally significant interaction, *F*(1, 119) = 3.88, *p* = .051. Post hoc tests failed to detect significant age differences in lure accuracy at either set size, both *p*_adjusted_ > .096.

Table 3 summarizes the model-based tests of our hypotheses (for age comparisons, see Table S1 in the online supplement). Among older adults, overloading WM at encoding led to a reduction in LTM estimates of gist retrieval (parameters *G*_*t*_ and *G*_*r*_), but we did not detect a credible set size effect on verbatim retrieval (parameter *V*_*t*_). Among young adults, we detected a credible set size effect on *G*_*r*_, indicating that young adults were less likely to retrieve a studied item’s gist in response to a lure if the item had been studied in SS6 sequences. We failed to detect credible set size effects on young adults’ estimates of *V*_*t*_ or *G*_*t*_. For the former, 0 was on the lower bound of the 95% CI and >95% of posterior samples were higher in the SS2 than SS6 condition, suggesting there could be an effect but that it is hard to detect even when the sample size was doubled relative to our predetermined sample size. Critically, evidence for an effect of WM set size on LTM verbatim retrieval was not central to our hypotheses.

**Table 3:** WM set size effects on LTM retrieval of specific and gist representations in Experiment 2

Parameter	Age	SS2	SS6	Δ(SS2 – SS6)	*p*(SS2 > SS6)
*V*_*t*_	Old	0.22 [0.02, 0.38]	0.15 [0.01, 0.29]	0.07 [−0.08, 0.20]	0.828
*G*_*t*_	Old	0.54 [0.37, 0.68]	0.35 [0.18, 0.49]	0.19 [0.04, 0.35]*	0.993
*V*_*r*_	Old	0.04 [0.00, 0.09]	= SS2	—	—
*G*_*r*_	Old	0.40 [0.32, 0.47]	0.33 [0.26, 0.40]	0.07 [0.02, 0.13]*	0.978
*V*_*t*_	Young	0.33 [0.09, 0.44]	0.22 [0.02, 0.33]	0.10 [−0.00, 0.20]	0.974
*G*_*t*_	Young	0.36 [0.21, 0.55]	0.29 [0.15, 0.47]	0.07 [−0.06, 0.19]	0.855
*V*_*r*_	Young	0.03 [0.00, 0.08]	= SS2	—	—
*G*_*r*_	Young	0.29 [0.22, 0.36]	0.23 [0.17, 0.29]	0.06 [0.01, 0.11]*	0.990

Multinomial-processing-tree (MPT) model-based estimates of long-term memory (LTM) specific/verbatim (*V*_*t*_ and *V*_*r*_) and gist (*G*_*t*_ and *G*_*r*_) retrieval in response to targets (*V*_*t*_ and *G*_*t*_) or related lures (*V*_*r*_ and *G*_*r*_) for items studied in working memory (WM) sequences of two (SS2) or six (SS6) items. Estimates are given as the group-level posterior means [95% Bayesian credible interval (CI)]. Estimates of the response bias parameters (see Fig. 2) were: (Older adults: *a* = 0.83 [0.68, 0.96], *b* = 0.55 [0.47, 0.63], *a*_*b*_ = 0.44 [0.34, 0.54]), (Younger adults: *a* = 0.70 [0.57, 0.87], *b* = 0.47 [0.43, 0.52], *a*_*b*_ = 0.26 [0.22, 0.30]). * denotes that the 95% CI of the posterior difference score Δ excluded 0. *p*(SS2 > SS6) gives the proportion of posterior samples for which the specified parameter was estimated to be higher in the SS2 than the SS6 condition

## General discussion

Retaining the meaning of past experiences is vital for successful comprehension (Kintsch & van Dijk, 1978) and allows individuals with more limited memory capabilities, like older adults, to adapt to these limitations (Greene & Naveh-Benjamin, 2024a). FTT posits that we can relatively automatically extract meaning (gist) of an experience in most situations, with substantially reduced need for attention compared to verbatim encoding (Brainerd & Reyna, 1990). Although studies of divided attention have largely supported this position (Greene & Naveh-Benjamin, 2023b; Odegard & Lampinen, 2005), recent evidence has cast doubt on the ubiquity of relatively automatic gist extraction (Greene et al., 2024; cf. Ricker & Hardman, 2017). When individuals encode more items than they can maintain concurrently in WM, their subsequent LTM retrieval of the gist of those items is disrupted. But is this an anomalous finding, or does it reflect a true boundary condition of FTT? Results of the present study show that this same pattern emerges among older adults, who preferentially rely on gist memory (Greene & Naveh-Benjamin, 2023a). As this pattern has now been detected in seven independent samples of young or older adults, it appears that it does reflect a true boundary where relatively automatic gist extraction breaks down. When attention is stretched too thinly across multiple to-be-encoded items, it limits our ability to encode durable gist representations of each item. Even if gist representations are initially established, they are vulnerable to interference under supra-capacity encoding. This could be attributed to increased compression of these events over the long-term, reducing the relative distinctiveness of items encoded in close succession, as predicted by temporal ratio models of memory (Brown et al., 2007).

But could relatively automatic gist extraction hold, even when WM is overloaded, if we deem it important to encode information in a way that will make it “stick” in LTM? Results of Experiment 2 show that, even under intentional learning instructions where participants knew their memory would be tested in the long-term, LTM gist retrieval was affected by the amount of information encoded concurrently into WM. To be clear, among young adults, there was some nuance to these results. When shown a target as recognition probes, young adults’ ability to retrieve the gist of this item from LTM was not credibly affected by the number of items encoded with it in WM. However, when shown a lure to a studied item, young adults, like older adults, were less capable of retrieving the studied item’s gist from LTM if the item was studied when WM was overloaded. From an applied perspective, these findings might warrant consideration of how much information is appropriate to present to students at once to minimize the risk of forgetting that information in the long-term.

The onus now falls on FTT, which has enjoyed enormous success in explaining sometimes counterintuitive findings in the memory and decision-making literatures (e.g., Brainerd & Reyna, 2002, 2015), to address why one of its core principles often falters in situations where individuals learn too much information at once. Clearly, this principle has been upheld in many other situations—under divided attention with a concurrent task (Odegard & Lampinen, 2005), in lexical decision tasks (Draine & Greenwald, 1998), and with speeded encoding (Greene & Naveh-Benjamin, 2024b). Thus, in many cases, it appears we can extract the meaning of an experience relatively automatically. But the present results highlight a boundary where relatively automatic gist extraction breaks down, inviting FTT to reconsider the ubiquity of this principle, and why it is sometimes violated. To be clear, FTT argues that automatic extraction is a sufficient but not necessary condition of gist encoding, and there are certainly other instances where gist encoding is effortful. For example, when the gist of an experience must be built up from connecting sequential elements (e.g., words in a sentence), then orienting attention toward peripheral features of the experience (e.g., checking for spelling errors) hampers gist extraction (Singer & Remillard, 2008). Our results complement these earlier findings while extending them to a domain in which item-specific gist is not contingent on comprehension of the preceding item in the sequence. Under these conditions, relatively automatic gist extraction has been the norm (e.g., Oliva & Torralba, 206; Potter, 1976; Tatler et al., 2003), but our results challenge that norm.

We are mindful that the present study is not without its limitations. The discrete-state assumption underlying the MPT model on which our inferences are based has been the subject of much controversy in the recognition literature (Pazzaglia et al., 2013), but we used this model because it is derived from FTT and thus is a powerful tool for testing FTT’s assumptions (Stahl & Klauer, 2008). There are also unresolved questions about the representativeness of online older adult samples (Greene & Naveh-Benjamin, 2022a). It is conceivable that our samples might be more highly educated than the general population of older adults, but if this is the case, then the effects reported here might *underestimate* the extent to which overloading WM impairs older adults’ LTM gist retrieval. Additionally, because our WM recognition tests were not suited to isolating gist from specific representations, we cannot determine whether overloading WM yielded immediate impairments in gist extraction or if the LTM effects stemmed from poorer consolidation of newly formed gist representations. The latter possibility seems to be anticipated by recent findings that, under conditions of high versus low cognitive load analogous to our supra- versus sub-capacity encoding conditions, there are immediate deficits in directly accessing the verbatim representations of items just-encoded into WM but not in reconstructing those items from their gist representations (Rosselet-Jordan et al., 2025). This suggests that gist representations might be initially formed under supra-capacity encoding conditions, but they are not effectively consolidated into more stable LTM traces.

Furthermore, although we have identified cases where overloading WM impairs LTM gist retrieval, it is possible there may be other situations where this does not occur. For instance, whether similar findings would be obtained with verbal stimuli, either presented visually or acoustically, is an unaddressed question. It is likely that an opposite effect would emerge in cases where one learns short (sub-capacity) versus long (supra-capacity) lists of related items that all share a global gist. Under these conditions, longer lists increase gist extraction because the same gist is encoded multiple times (Brainerd et al., 2001; Robinson & Roediger, 1997). Moreover, our intentional LTM encoding manipulation in Experiment 2 did not substantially improve LTM recognition performance over conditions of more incidental encoding in Experiment 1. It is possible that a stronger manipulation could attenuate the effect of WM encoding load on subsequent LTM gist retrieval, though such a manipulation could alter WM performance by encouraging the use of long-term learning strategies not commonly employed in WM tasks. Even if different effects emerged with semantically associated or categorized lists, the fact that we consistently observe effects of supra-capacity encoding on LTM gist retrieval for familiar objects indicates that there is at least some boundary condition on relatively automatic gist extraction.

Despite these limitations, the present study has more clearly mapped out the boundaries under which relatively automatic gist extraction breaks down. The fact that such a boundary exists at all places critical constraints on leading memory theories like FTT. These kinds of theory-defying constraints have had transformative impacts throughout the history of science. We hope that they will similarly yield a more comprehensive understanding of when we can and when we cannot automatically extract the meaning of experiences for long-term retention.

## Supplementary Information

Below is the link to the electronic supplementary material.

Supplementary file1 (DOCX 410 KB)

## Data Availability

All study materials are publicly available (https://bradylab.ucsd.edu/). All deidentified data available: https://osf.io/b6qa2/.

## Footnotes

1 Throughout, we define gist encoding as “relatively automatic” in the sense that it is presumed to be *more* automatic than verbatim encoding. Some phases of encoding might be attention-demanding for both gist and verbatim encoding, whereas other (e.g., later) phases might be attention-demanding only for verbatim encoding (cf. Greene & Naveh-Benjamin, 2024b).

2 We also replicated Greene et al.’s Experiment 2 in a separate sample of older adults. Results of this second experiment replicate those reported here (see online supplement).

3 Occasional fluctuations in attention could explain why *k* was not exactly two items in the SS2 condition.

4 We accidentally posted one additional slot on Prolific, so the final sample of young adults was 81.

## Code availability

Analysis scripts can be found at: https://osf.io/b6qa2/.
