# Supplementary material for: Long-term representational costs of overloading working memory
Source: Psychon Bull Rev. 2026 Feb 19;33(2):87. doi: 10.3758/s13423-025-02826-y (PMC12920783; doi:10.3758/s13423-025-02826-y)
Supplement: Supplementary file 1 — Supplementary file1 (DOCX 410 KB) [file 13423_2025_2826_MOESM1_ESM.docx]

**Supplemental Materials for**

Greene, N. R., Guitard, D., Forsberg, A., Cowan, N., & Naveh-Benjamin, M. *Long-Term Representational Costs of Overloading Working Memory.*

**Sample Size Determination**

An *n* of 40 matches the sample sizes of each experiment in Greene et al. (2024), which were reported to be well-powered (89% true discovery rate according to a Bayes Factor Design Analysis) to detect a medium-sized effect (*d* ≈ 0.70, ranging from 0.60 to 0.80) between two conditions, such as an effect of SS6 versus SS2 on LTM accuracy. Complete details can be found in Greene et al. (2024). We also conducted an *a priori* power analysis in the multiTree program (Moshagen, 2010) to determine the minimum sample size needed to detect an effect of the same size as the WM set size effect (Δ = SS2 – SS6) on LTM gist retrieval obtained in Greene et al. (2024) Exp. 1b. We set the expected effect size equal to that experiment’s group-level posterior mean difference (μ(Δ)) in MPT model estimates of *G_t_* (μ(Δ*G_t_*) = 0.26) and *G_r_* (μ(Δ*G_r_*) = 0.11). The minimum total number of LTM recognition responses across *all* participants needed to detect effects of this size in the present study, with 80% power at α = .05, was 3,439.29 observations. With 216 LTM recognition trials per participant in the present experiment, this amounts to a minimum sample size of 16 participants. To account for the uncertainty in the true effect size Δ conveyed by the hierarchical Bayesian model, we additionally conducted a *post hoc* power analysis (Moshagen, 2010) to detect the minimum Δ we could expect to detect at a chosen *n* = 40 participants, each completing 216 trials. With *n* = 40, we could expect to detect a group-level Δ = 0.09 on both gist parameters with 86.57% power at α = 0.05.

**Estimating WM Capacity**

We used hierarchical Bayesian modeling to estimate group- and individual-level WM capacities *k*. Given a set of *N* items, *k* of those items can be maintained concurrently in WM. The probability that an old item selected as a test probe currently resides in WM is *k*/*N*, such that if *k* ≥ *N*, the participant will perfectly detect any old item in an immediate recognition test. Cowan et al. (2013, Appendix, Model 2) presented an equation to solve for *k* based on the observed proportions of correct rejections (*cr*) and false alarms (*f*) to new items in single-probe procedures like ours: *k* = *N* × (*cr* – *f*)/*cr*. However, this equation can give non-sensical negative estimates of *k* if *f* > *cr*. Hierarchical Bayesian modeling circumvents this issue by specifying weakly informative priors that can constrain *k* ≥ 0. We followed the approach outlined in Greene et al. (2024) for fitting the hierarchical Bayesian WM capacity model to the data.

**MPT Model Estimation Procedures**

Posterior summaries of model parameters were estimated under a latent-trait prior specification of the hierarchical Bayesian MPT model. The latent-trait approach (Klauer, 2010) models the MPT parameter vector **θ** for participant *j* (**θ**_j_) on a latent-probit scale, Φ^-1^(**θ**_j_), where each **θ**_j_ is a realization from a multivariate normal distribution, **θ**_j_ ~ ***N***(**μ**, **Σ**). Hyperpriors on group-level parameters **μ** (group means of each parameter) and **Σ** (group-level parameter covariances) minimize extreme values of **θ**_j_ by shrinking individual-level estimates toward group-level estimates. We retained the default hyperprior specifications for **μ** and **Σ** from the TreeBUGS package (Heck et al., 2018), as these were chosen to be weakly informative. To sample from the posterior distribution of each parameter, we specified three independent Markov chain Monte Carlo (MCMC) chains, each 15,000 adaptation iterations, 10,000 burn-in iterations, and 50,000 sampling iterations, with every 10^th^ iteration retained to speed computing time. Between-chain convergence on a stable posterior distribution was monitored by the $\hat{R}$ statistic and was deemed satisfactory when $\hat{R}$ < 1.05, which was satisfied for all parameters in each experiment.

The full MPT model contains 11 free parameters ($V_{t}^{SS2}$, $V_{t}^{SS6}$, $V_{r}^{SS2}$, $V_{r}^{SS6}$, $G_{t}^{SS2}$, $G_{t}^{SS6}$, $G_{r}^{SS2}$, $G_{r}^{SS6}$, *a*, *b*, and *a_b_*), but with five memory probe types (old items from each set size, lures for each set size, and novel foils), each with three response options, there were only 10 experimental degrees of freedom. Thus, at least one equality constraint is required to make the model identified. Initially, we constrained *a_b_* = *a*, but this model yielded an unsatisfactory fit to the data. We removed this constraint and instead set $V_{r}^{SS2}$ = $V_{r}^{SS6}$ as estimates of verbatim retrieval in response to lures was consistently near 0, regardless of set size. This modified model yielded satisfactory posterior-predictive fit in all experiments. Fit was evaluated by simulating 1,000 hypothetical datasets from the posterior distributions of the model parameters and then comparing the posterior-predicted and observed mean LTM recognition response frequencies with the *T_1_* statistic (Klauer, 2010) and its associated posterior predictive *p* (PPP) value. Model fit was considered satisfactory if PPP > 0.05, which was satisfied in all experiments, all PPP > 0.358.

**Age Group Differences**

Tests of age group differences were not germane to our hypotheses. However, we carried out exploratory tests of these differences on the MPT model-based estimates of specific and gist memory retrieval. To do so, in each experiment, we fit the model to the combined data of young and older adults and regressed each parameter on an effect-coded Age predictor (-1 = young, 1 = older adult), with a weakly-informative standard normal prior on each slope. We used the Savage-Dickey method (Wagenmakers et al., 2010) to derive Bayes factors (*BF*) for whether the slope of Age (*β_Age_*) on each MPT model parameter differed from 0. If the posterior density at 0 for a given slope parameter has increased (decreased) relative to its prior density at 0, the resulting *BF* will provide evidence for (against) a null effect.

Table S1 lists the resulting *BF*s for the effect hypothesis that *β_Age_* ≠ 0 (*BF*_10_) and the null hypothesis that *β_Age_* = 0 (*BF*_01_) for each parameter in both experiments. A *BF* > 3 indicates moderate or greater support for the respective hypothesis. In most cases, the null hypothesis was favored (most *BF*_01_ > 3). However, the evidence was only weakly in favor of the null hypothesis on parameters *G_t_* (SS6) and *V_r_* in Experiment 1 and on parameters *G_t_* (SS2), *G_r_* (SS2), and *G_r_* (SS6) in Experiment 2. Meanwhile, in Experiment 1, older adults’ estimates of gist retrieval in response to lures (parameter *G_r_*) at both set sizes were *higher* than the corresponding estimates of these parameters from the young adults in Exp. 1b of Greene et al. (2024). That is, when older adults failed to retrieve a studied item’s verbatim representation in response to a lure, they were nonetheless *more* successful than younger adults, under the same conditions, at retrieving that item’s gist, regardless of the set size from which the item came. However, as reported in Table 1 in the main text, overloading WM exerted a similar effect on *G_r_* among young and older adults alike (i.e., $G_{r}^{SS6}<G_{r}^{SS2}$). This suggests the effect of overloading WM at encoding on LTM gist retrieval can be found across groups that differ in their overall reliance on gist memory.

**Table S1.** *Hypothesis Tests for Age Effects on Each MPT Parameter*

|  | Experiment 1 | | Experiment 2 | |
| --- | --- | --- | --- | --- |
| Parameter | *BF*_10_ | *BF*_01_ | *BF*_10_ | *BF*_01_ |
| *V_t_* (SS2) | 0.195 | **5.134** | 0.185 | **5.392** |
| *V_t_* (SS6) | 0.107 | **9.322** | 0.138 | **7.271** |
| *G_t_* (SS2) | 0.135 | **7.428** | 0.906 | **1.103** |
| *G_t_* (SS6) | 0.844 | **1.186** | 0.089 | **11.243** |
| *V_r_* | 0.366 | **2.732** | 0.320 | **3.126** |
| *G_r_* (SS2) | **8.992** | 0.111 | 0.706 | **1.416** |
| *G_r_* (SS6) | **5.581** | 0.179 | 0.870 | **1.149** |
| *a* | 0.117 | **8.556** | 0.068 | **14.667** |
| *b* | 0.127 | **7.873** | 0.036 | **27.554** |
| *a_b_* | 0.076 | **13.171** | 0.112 | **8.357** |

*Note.* Hypothesis tests evaluate whether the slope (β_Age_) of the effect-coded (-1 = young, 1 = older adult) age predictor variable differed from 0 using the Savage-Dickey method. *BF*_10_ and *BF*_01_ give the resulting Bayes factor for the effects and null hypothesis, respectively. Bolded entry corresponds to the hypothesis with stronger support based on the data and weakly informative priors. Experiment 1 compares older adults from the present study (Experiment 1) with the young adults from Experiment 1b of Greene et al. (2024).

**Experiment 1b: Simultaneous Presentation**

We replicated the procedure of Experiment 1 in an independent sample of 40 older adults (18 females, 22 males) recruited from Prolific using the same eligibility criteria, with the additional constraint that participants were ineligible if they participated in the previous experiments. Participants were aged between 65 and 80 (*M* = 69.65, *SD* = 4.69). A plurality of participants had either a technical/Associate’s degree (22.5%) or a Bachelor’s degree (22.5%), followed by a high-school diploma (20%), a Master’s degree (15%), a GED (10%), or less than a high-school degree (10%). The only difference in the procedure was that, during encoding, objects were presented simultaneously rather than sequentially (cf., Greene et al. (2024), Exp. 2). Each object appeared in one of eight random locations, spaced equidistantly from the center of the screen. The display was presented for 250ms × *n* items (i.e., 500ms in the SS2 condition, and 1500ms in the SS6 condition). All other procedures were identical to Experiment 1.

WM and LTM recognition results appear in Figure S1. In the WM tests, older adults exhibited poorer discrimination (i.e., lower *A'*) in the SS6 condition (*M* = 0.83, *SD* = 0.11) than the SS2 condition (*M* = 0.97, *SD* = 0.04), *t*(39) = 7.61, *p* < .001. Their WM was also overloaded by almost three items in the SS6 condition, group-level posterior mean of *k* = 3.19, 95% CI [2.82, 3.56], whereas in the SS2 condition, they could typically maintain both items in WM, *k* = 1.81, 95% CI [1.74, 1.86]. Meanwhile, in the LTM tests, older adults were more accurate at identifying targets as “old” if they came from SS2 arrays (*M* = 0.55, *SD* = 0.03) rather than SS6 arrays (*M* = 0.40, *SD* = 0.03), *t*(39) = 7.99, *p* < .001. However, we did not detect a significant set size effect on LTM recognition accuracy for similar lures, *t*(39) = -0.20, *p* = .841, but older adults were more likely to erroneously call lures “old” for SS2 lures (*M* = 0.43, *SD* = 0.03) than SS6 lures (*M* = 0.32, *SD* = 0.03), *t*(39) = 4.84, *p* < .001. As listed in Table S2, overloading WM during encoding led to reduced LTM retrieval of gist representations of studied items whether those items (targets) or lures appeared as test probes.

**Figure S1.** *WM and LTM Recognition Results in Experiment 1b*


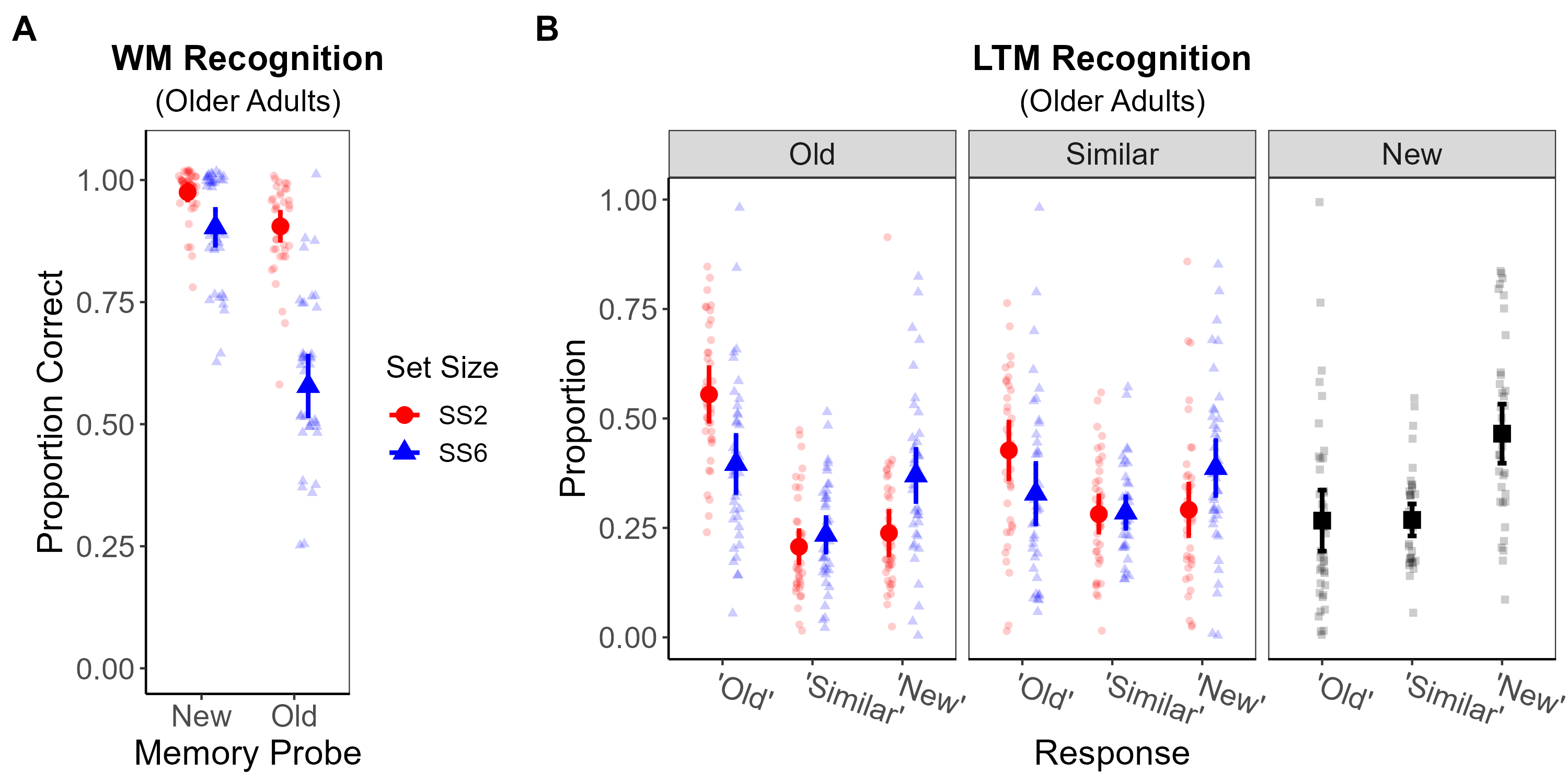


*Note.* (A) Proportion of correct responses to old and new probes in the working memory (WM) recognition tests for set size two (SS2) and set size six (SS6) arrays in Experiment 1b. (B) Proportions of “old”, “similar,” and “new/different” responses to old, similar, and new probes in the long-term memory (LTM) recognition tests of Experiment 1b. Responses to old items and similar lures broken down by set size condition. In both panels, group means appear as large, bold shapes, with individual participants’ means underlaid as faded shapes. Error bars represent 95% confidence intervals of the means.

**Table S2.** *WM Set Size Effects on LTM Retrieval of Specific and Gist Representations in Experiment 1b*

| Parameter | SS2 | SS6 | Δ(SS2–SS6) | *p*(SS2>SS6) |
| --- | --- | --- | --- | --- |
| *V_t_* | 0.15 [0.01, 0.32] | 0.10 [0.01, 0.19] | 0.05 [-0.11, 0.22] | 0.713 |
| *G_t_* | 0.36 [0.14, 0.52] | 0.09 [0.01, 0.21] | 0.26 [0.05, 0.44]* | 0.991 |
| *V_r_* | 0.03 [0.00, 0.07] | = SS2 | ------ | ------ |
| *G_r_* | 0.34 [0.26, 0.43] | 0.12 [0.06, 0.19] | 0.22 [0.13, 0.30]* | 1.00 |

*Note.* MPT model-based estimates of LTM specific/verbatim (*V_t_* and *V_r_*) and gist (*G_t_* and *G_r_*) retrieval in response to targets (*V_t_* and *G_t_*) or related lures (*V_r_* and *G_r_*) for items studied WM arrays of two (SS2) or six (SS6) items concurrently in Experiment 1b. Estimates are given as the group-level posterior means [95% Bayesian credible interval (CI)]. Estimates of the response bias parameters (see Figure 2 main text) were: *a* = 0.83 [0.68, 0.96], *b* = 0.44 [0.34, 0.54], *a_b_* = 0.55 [0.47, 0.63]. * denotes that the 95% CI of the posterior difference score Δ excluded 0. *p*(SS2>SS6) gives the proportion of posterior samples for which the specified parameter was estimated to be higher in the SS2 than the SS6 condition.

**Experiment 2 MPT Results for Original 40 Young Adults**

Table S3 lists the MPT results in Experiment 2 for the original 40 young adults We failed to detect credible set size effects on any parameter (all 95% CI’s of Δ encompassed 0). However, failing to detect an effect is not equivalent to detecting a null effect. The effect might be much smaller than anticipated and thus requires a large sample to detect. As optional stopping does not bias the results in the Bayesian statistical framework (Rouder, 2014), we opted to double the sample size in the young adult group to see if evidence for an effect would emerge (see main text).

**Table S3.** *MPT Results in Experiment 2 for the Initial 40 Young Adults*

| Parameter | SS2 | SS6 | Δ(SS2–SS6) | *p*(SS2>SS6) |
| --- | --- | --- | --- | --- |
| *V_t_* | 0.27 [0.04, 0.45] | 0.17 [0.01, 0.33] | 0.10 [-0.06, 0.22] | 0.908 |
| *G_t_* | 0.41 [0.21, 0.59] | 0.33 [0.15, 0.50] | 0.08 [-0.08, 0.24] | 0.845 |
| *V_r_* | 0.06 [0.00, 0.13] | = SS2 | ------ | ------ |
| *G_r_* | 0.28 [0.20, 0.38] | 0.24 [0.17, 0.33] | 0.04 [-0.03, 0.11] | 0.865 |

*Note.* See Table S2 caption for details. Estimates of the response bias parameters in the initial sample of 40 young adults were: *a* = 0.71 [0.51, 0.87], *b* = 0.43 [0.36, 0.49], *a_b_* = 0.22 [0.15, 0.28].

**References**

Cowan, N., Blume, C. L., & Saults, J. S. (2013). Attention to attributes and objects in working memory. *Journal of experimental psychology. Learning, memory, and cognition*, *39*(3), 731–747. <https://doi.org/10.1037/a0029687>

Greene, N. R., Guitard, D., Forsberg, A., Cowan, N., & Naveh-Benjamin, M. (2024). Working memory limitations constrain visual episodic long-term memory at both specific and gist levels of representation. *Memory & Cognition, 52*, 1958-1982. <https://doi.org/10.3758/s13421-024-01593-w>

Heck, D. W., Arnold, N. R., & Arnold, D. (2018). TreeBUGS: An R package for hierarchical multinomial-processing-tree modeling. *Behavior Research Methods, 50*(1), 264-284. <https://doi.org/10.3758/s13428-017-0869-7>

Klauer, K. C. (2010). Hierarchical multinomial processing tree models: A latent-trait approach. *Psychometrika, 75*, 70-98. <https://doi.org/10.1007/s11336-009-9141-0>

Moshagen, M. (2010). multiTree: A computer program for the analysis of multinomial processing tree models. *Behavior Research Methods, 42*(1), 42–54. [https://doi.org/10.3758/BRM.42.1.42](https://psycnet.apa.org/doi/10.3758/BRM.42.1.42)

Wagenmakers, E.-J., Lodewyckz, T., Kuriyal, H., & Grasman, R. (2010). Bayesian hypothesis testing for psychologists: A tutorial on the Savage-Dickey method. *Cognitive Psychology, 60*(3), 158-189. <https://doi.org/10.1016/j.cogpsych.2009.12.001>
